# The role of affective temperaments in predicting depression and anxiety symptoms in patients with primary hyperparathyroidism

**DOI:** 10.1371/journal.pone.0339321

**Published:** 2025-12-26

**Authors:** Deniz Alçı, Mustafa Eroğlu, Mehmet Aşık

**Affiliations:** 1 Department of Psychiatry, Faculty of Medicine, Balıkesir University, Balıkesir, Türkiye; 2 Department of Internal Medicine, Division of Endocrinology, Diabetes and Metabolism, Faculty of Medicine, Balıkesir University, Balıkesir, Türkiye; 3 Department of Endocrinology, Diabetes and Metabolism, Bodrum American Hospital, Muğla, Türkiye; Oita University Faculty of Medicine, JAPAN

## Abstract

**Background:**

This study aimed to investigate the relationship between affective temperament traits and the severity of depression and anxiety symptoms in patients diagnosed with primary hyperparathyroidism.

**Methods:**

A cross-sectional study was conducted including 47 patients with primary hyperparathyroidism and 36 healthy controls. Participants were evaluated using the Memphis, Pisa, Paris and San Diego Temperament Assessment Scale to assess affective temperament profiles, and the Hospital Anxiety and Depression Scale to determine symptoms of anxiety and depression. Clinical, biochemical, and sociodemographic data were also collected. Correlation analyses and a generalized linear model were used to explore associations and predictors of psychiatric symptoms. Data were collected at Çanakkale Onsekiz Mart University between June 2016 and January 2017.

**Results:**

Patients with primary hyperparathyroidism showed significantly higher scores for depressive, cyclothymic, and anxious temperaments compared to healthy controls. Anxiety and depression scores were also significantly higher in the patient group. Among patients, depressive, cyclothymic, irritable, and anxious temperament traits were positively correlated with both anxiety and depression levels, whereas hyperthymic temperament showed no significant association. Multivariate analysis revealed that anxious and cyclothymic temperaments were significant predictors of anxiety symptoms, while hyperthymic temperament was associated with lower depression scores. No significant associations were found between biochemical parameters and psychiatric symptom severity, except for a positive correlation between serum calcium levels and hyperthymic temperament.

**Conclusions:**

Affective temperament characteristics, particularly anxious and cyclothymic traits, are closely associated with the severity of anxiety and depression symptoms in patients with primary hyperparathyroidism. Hyperthymic temperament may act as a protective factor against depression in this population. Incorporating temperament assessment into the clinical evaluation of these patients may facilitate early identification of those at higher risk for psychiatric comorbidities and guide more effective, individualized intervention strategies.

## Introduction

Primary hyperparathyroidism (PHPT) is traditionally characterised by the symptoms encapsulated in the phrase “stones, bones, abdominal groans, and psychic moans,” resulting from hypercalcemia [1]. While psychiatric symptoms are termed “psychic moans” in this context, depression and anxiety in patients with primary hyperparathyroidism are usually considered “non-classic” manifestations [2,3]. Research conducted over the past decade has demonstrated that depression and anxiety are notably prevalent among people diagnosed with PHPT. Recent studies have shown that depression and anxiety are common in individuals with primary hyperparathyroidism. Approximately 30% of patients experience depression and up to 49% have anxiety symptoms [3]. A recent review found that among those scheduled for parathyroidectomy, 43–53% had symptoms of anxiety and 32–62% showed signs of depression [4]. This variability is largely attributable to differences in assessment instruments. For example, depression prevalence is 10% according to ICD-10 criteria, but can reach 60% in studies using symptom scales [5]. However, it is possible to assert that clinically substantial psychiatric symptomatology is present in one of every three to four patients with PHPT [1]. Depression and anxiety are the predominant psychiatric manifestations of primary hyperparathyroidism (PHPT) and have been associated with a range of symptoms, including exhaustion, sleep disturbances, and psychosis [6,7]. The high prevalence of these comorbidities is clinically significant, as depressive and anxious symptoms reduce patients’ quality of life, negatively affect their well-being, and increase the overall disease burden [8–10]. The presence of depression might complicate the management of complications associated with primary hyperparathyroidism, such as osteoporosis and nephrolithiasis, and elevate the use of healthcare services [11,12].

Recognition and effective management of psychiatric comorbidities are essential in the comprehensive treatment of PHPT. However, identifying symptoms of depression and anxiety can be challenging, as they may be attributed to other causes or may appear mild [2,13,14]. Consequently, regular monitoring for depression and anxiety is advised for patients with PHPT [15,16]. Psychiatric symptoms in PHPT, especially depression and anxiety, can significantly impair functioning in family, social, and occupational domains, resulting in notable reductions in quality of life and daily performance [2]. Depression is also associated with an increased risk of suicidal ideation. In a large prospective study, the rate of suicidal ideation decreased from 22% before surgery to 10.7% one year after parathyroidectomy [2,17]. Moreover, depression itself may mask somatic symptoms of PHPT and discourage patients from recognizing physical complaints or seeking timely medical evaluation [18]. These observations underscore that the neuropsychiatric effects of primary hyperparathyroidism are at least partially reversible, as evidenced by significant improvement in depression, anxiety, and functional outcomes after parathyroidectomy [19]. However, current guidelines do not list psychiatric symptoms as standalone surgical indications in the absence of standard criteria [20]. Consequently, psychiatric symptoms associated with PHPT must be accurately assessed, and patients should be referred to appropriate psychiatric care and support as part of an integrated treatment strategy.

The mechanisms underlying the neuropsychiatric symptoms of primary hyperparathyroidism (PHPT) remain inadequately understood; nonetheless, the direct impacts of hypercalcemia and parathyroid hormone (PTH) on the central nervous system are significant. Elevated serum calcium concentrations may result in diminished motivation, confusion, depressive symptoms, and anxiety [21]. Severe hypercalcemia can lead to acute delirium, psychosis, and coma. Long-term depression and alterations in personality have been observed, even in instances of mild to moderate hypercalcemia [17]. Excess parathyroid hormone (PTH) and elevated calcium levels may interfere with the metabolism of monoamine neurotransmitters, potentially impacting mood-regulating neurotransmitters like dopamine and serotonin [22]. PTH is believed to induce neuroinflammation through the modulation of cytokine regulation in the central nervous system [23].

Nevertheless, depression and anxiety are not solely influenced by biochemical factors; they are also influenced by the inherent and lifelong temperamental characteristics of the individual [24]. The association between affective temperament and psychiatric conditions, including anxiety and depression, is well-established in the field of psychiatry [25–27]. Individuals exhibiting depressive, cyclothymic, and anxious temperaments are at an increased risk of developing depression and anxiety when confronted with stress or medical conditions [27]. Individuals with hyperthymic temperaments might have more resistance to these symptoms [26,28]. The influence of temperament on psychiatric symptoms in chronic diseases like PHPT may significantly impact patient management. The literature also supports the potential of affective temperament characteristics to predict the risk of future psychiatric disorders [29–31]. The assessment of temperament in patients with primary hyperparathyroidism (PHPT) could serve as an important tool to evaluate the risk of depression and anxiety, facilitating a multidisciplinary approach in patient management.

Therefore, in this study, we aimed to examine the relationship between affective temperament characteristics and the severity of depression and anxiety symptoms in patients with primary hyperparathyroidism. Assessing temperament may enhance risk stratification for psychiatric comorbidity and facilitate more comprehensive and individualized management strategies in this patient group. Given the high prevalence and clinical impact of psychiatric symptoms in PHPT and the lack of research examining the role of stable affective dispositions in this population, identifying temperament patterns that may elevate or reduce psychiatric vulnerability constitutes an important clinical need.

## Methods

### Study design and participants

This study was designed as a cross-sectional study including patients aged 18 years or olderdiagnosed with primary hyperparathyroidism (PHPT) alongside a healthy control group. The patient group consisted of individuals followed in the endocrinology clinic of a university hospital who were either newly diagnosed or previously diagnosed but had not yet received surgical or medical treatment. The diagnosis of primary hyperparathyroidism was based on elevated serum parathyroid hormone (PTH) and calcium levels above the reference range, and was confirmed by endocrinological assessment.

The control group consisted of healthy individuals who visited the endocrinology clinic for general health screening, had no chronic diseases or psychiatric diagnoses, and had normal thyroid and parathyroid function test results. Although the two groups differed in age, both had a similar gender distribution.

Inclusion criteria:

- Age ≥ 18
- Biochemically confirmed primary hyperparathyroidism
- Not yet treated surgically or medically
- Consent to participate

Exclusion criteria:

- Current psychiatric diagnosis or psychiatric medication
- Neurological disorder or cognitive impairment
- Substance abuse history
- Severe systemic illness,

Participants who met any of the exclusion criteria were not included in the study. The study protocol was approved by the Clinical Research Ethics Committee of Çanakkale Onsekiz Mart University (approval number: 2015−11, date: 21/07/2015). Written informed consent was obtained from all participants. Data were collected at Çanakkale Onsekiz Mart University between June 2016 and January 2017. The study did not include minors. Informed consent was obtained by the attending endocrinologist during the clinical evaluation, and the process was witnessed by the nurse and medical interns present at the examination.

### Assessment tools

Memphis, Pisa, Paris and San Diego Temperament Assessment Scale (TEMPS-A):

TEMPS-A scale was developed by Akiskal et al. to assess affective temperament profiles [32]. The Turkish adaptation of the TEMPS-A has demonstrated strong psychometric properties. In the validation study by Vahip et al., Cronbach’s alpha coefficients were 0.77 for the depressive, 0.85 for the cyclothymic, 0.80 for the hyperthymic, 0.82 for the irritable, and 0.84 for the anxious subscales, with test–retest correlations ranging from 0.73 to 0.93 [33]. The instrument comprises five subscales measuring depressive, cyclothymic, hyperthymic, irritable, and anxious temperament traits.

### Hospital anxiety and depression scale (HADS)

Anxiety and depression symptoms were assessed using the Hospital Anxiety and Depression Scale (HADS), developed by Zigmond and Snaith [34]. The Turkish version of the Hospital Anxiety and Depression Scale similarly demonstrated good reliability, with Cronbach’s alpha values of 0.85 (Anxiety) and 0.77 (Depression) in the original validation study by Aydemir et al. [35] The instrument comprises 14 items and yields separate scores for anxiety and depression. A score of 0–7 is considered normal, 8–10 is classified as borderline, and a score of 11 or higher indicates clinically significant symptoms.

### Clinical and sociodemographic data

Data on participants’ age, gender, body mass index (BMI), smoking status, existing chronic diseases, medications, and biochemical parameters (TSH, PTH, serum calcium levels) were systematically documented using a structured form.

### Statistical analysis

The data obtained in this study were analyzed using IBM SPSS Statistics 22. The Shapiro-Wilk test was conducted to assess the normality of the data distribution, alongside the examination of Q-Q plots and histograms.

Descriptive statistics involved the calculation of criteria including minimum, maximum, mean, standard deviation, as well as frequencies and percentages to illustrate data distribution. The Mann-Whitney U test was used to compare numerical variables between two groups, and the Kruskal-Wallis test was used to compare numerical variables among more than two groups. The Pearson Chi-Square test was used to compare categorical variables between groups, and the Yates-corrected Chi-Square test was applied when appropriate. The internal consistency of the scales was evaluated by calculating Cronbach’s alpha coefficient. Spearman’s rho correlation analysis was performed to assess the relationships between the scales.

A Generalized Linear Model (GLM) with a Gaussian (normal) distribution and identity link function was used for continuous dependent variables. Backward variable selection was applied, and the model with the lowest Akaike Information Criterion (AIC) and Bayesian Information Criterion (BIC) values was selected.

The required sample size to detect a moderate effect (Cohen’s d = 0.6) with 80% power at a significance level of 0.05 was calculated using G*Power software. At least 36 participants per group were needed. The study included 47 patients and 36 controls, which was sufficient for statistical power.

Since the PHPT and control groups differed significantly in age, we performed additional age-adjusted generalized linear models (GLM) for all HADS and TEMPS-A subscales. Each model included group as the main predictor and age as a covariate. These analyses were conducted to account for the potential confounding effect of age on temperament traits and psychiatric symptom severity.

### Ethics statement

The study was approved by the Clinical Research Ethics Committee of Çanakkale Onsekiz Mart University (Approval No: 2015−11, Date: 21/07/2015). Written informed consent was obtained from all participants prior to data collection.

## Results

This study investigated the impact of affective temperament traits on anxiety and depression in patients with primary hyperparathyroidism (PHPT). Of the total 83 participants, 47 (56.6%) were in the PHPT group and 36 (43.4%) were in the healthy control group.

### Demographic and clinical characteristics

There was a significant difference in mean age between the groups (p < 0.001), with the PHPT group being older (51.91 ± 13.18 years) than the control group (35.50 ± 8.82 years) (see Table 1). Most participants were female (85.5%), and the gender distribution was similar in both groups (p > 0.05).

**Table 1:** Demographic and clinical characteristics of participants.

General Characteristics		PHPT Patients (n = 47)	Controls (n = 36)	Total (N = 83)	t/ χ2	p
Age (years)	Min–Max	19–74	23–55	19–74	16.443	<0.001*
	Mean ± SD	51.91 ± 13.18	35.50 ± 8.82	44.80 ± 14.06		
Age group	≤45 years	12 (25.5%)	30 (83.3%)	42 (50.6%)	24.984	<0.001*
	>45 years	35 (74.5%)	6 (16.7%)	41 (49.4%)		
Gender	Female	42 (89.4%)	29 (80.6%)	71 (85.5%)	0.665	0.415
	Male	5 (10.6%)	7 (19.4%)	12 (14.5%)		

Quantitative data are presented as minimum–maximum and mean ± standard deviation; qualitative data as n (%). t: Student t-test; χ²: Yates-corrected chi-square test. *p < 0.01.

Among the PHPT group, hypertension was present in 21.3%, diabetes in 6.4%, and coronary artery disease in 2.1% (see Table 2). The mean TSH level in the PHPT group was 1.50 ± 0.64 mIU/L (n = 28), the mean PTH level was 167.04 ± 147.84 pg/mL, and the mean serum calcium level was 11.03 ± 0.99 mg/dL.

**Table 2:** Clinical and biochemical characteristics of the PHPT patient group.

Clinical/biochemical variables		n (%)/ Mean/ Min-Max
History of CAD (Coronary artery disease)	Yes	1 (2.1)
	No	46 (97.9)
History of HT (Hypertension)	Yes	10 (21.3)
	No	37 (78.7)
History of DM (Diabetes Mellitus)	Yes	3 (6.4)
	No	44 (93.6)
Other diseases	Yes	6 (12.8)
	No	41 (87.2)
Medication use	Yes	16 (34.0)
	No	31 (66.0)
TSH (mIU/L)	Min–Max	0.01-199.00
	Mean ± SD (Median)	11.056 ± 34.84 (1.45)
PTH (pg/mL)	Min–Max	21.90-848.00
	Mean ± SD (Median)	167.04 ± 147.84 (127)
Calcium (mg/dL)	Min–Max	9.20-12.84
	Mean ± SD (Median)	11.03 ± 0.99 (11.05)

**Data are presented as minimum–maximum, mean ± standard deviation (median), or number (percentage) as appropriate. Temperament Characteristics and Levels of Depression and Anxiety.**

The mean depressive temperament score (TEMPS-A) was 9.45 ± 3.98 (median 10) in the PHPT group and 4.86 ± 3.03 (median 4) in the control group; this difference was statistically significant (p < 0.001) (see Table 3). The cyclothymic temperament score was 9.64 ± 4.87 (median 9) in the PHPT group and 5.92 ± 4.14 (median 5) in the control group (p = 0.001) (see Table 3). The mean anxious temperament score was 10.96 ± 6.54 (median 11) in the PHPT group and 5.97 ± 5.42 (median 4) in the control group; this difference was statistically significant (p < 0.001).

**Table 3:** Comparison of TEMPS-A and HADS scores between PHPT patients and controls.

Scale	Subscale	PHPT (n = 47) Mean ± SD (Median)	Control (n = 36) Mean ± SD (Median)	Z	p
TEMPS-A	Depressive	9.45 ± 3.98 (10)	4.86 ± 3.03 (4)	−5.061	<0.001**
	Cyclothymic	9.64 ± 4.87 (9)	5.92 ± 4.14 (5)	−3.416	0.001**
	Hyperthymic	9.26 ± 4.20 (10)	10.11 ± 4.6 (9.5)	−0.599	0.549
	Irritable	5.06 ± 4.61 (4)	2.97 ± 2.72 (2)	−1.792	0.073
	Anxious	10.96 ± 6.54 (11)	5.97 ± 5.42 (4)	−3.499	<0.001**
HADS	Anxiety	9.00 ± 4.09 (9)	1.94 ± 0.23 (2)	−7.455	<0.001**
	Depression	7.70 ± 4.30 (8)	5.39 ± 3.20 (5)	−2.643	0.008**

Data are presented as mean ± standard deviation (median). Z: Mann-Whitney U test; *p < 0.05; **p < 0.01*.

To address the significant age difference between groups, additional generalized linear models (GLM) were computed including age as a covariate. After adjusting for age, PHPT patients continued to show significantly higher depressive temperament (β = –5.09, p < 0.001), cyclothymic temperament (β = –3.79, p = 0.003), irritable temperament (β = –3.67, p = 0.001), and anxious temperament (β = –6.27, p < 0.001) scores compared with controls. Hyperthymic temperament remained non-significantly different (p = 0.46). The difference in HADS anxiety scores also remained highly significant after age adjustment (β = –7.84, p < 0.001), whereas the group difference in HADS depression scores was no longer significant (β = –2.09, p = 0.052). These results indicate that the observed temperament and anxiety differences between groups are not explained by age.

There was no significant difference between groups in hyperthymic temperament scores: 9.26 ± 4.20 (median 10) in the PHPT group and 10.11 ± 4.60 (median 9.5) in controls (p = 0.549). The mean irritable temperament score was 5.06 ± 4.61 (median 4) in the PHPT group and 2.97 ± 2.72 (median 2) in the control group; this difference was not statistically significant (p = 0.073).

The HADS anxiety score was 9.00 ± 4.09 (median 9) in the PHPT group and 1.94 ± 0.23 (median 2) in controls (p < 0.001). HADS depression scores were 7.70 ± 4.30 (median 8) in the PHPT group and 5.39 ± 3.20 (median 5) in the control group (p = 0.008).

### The link between temperament traits and levels of anxiety and depression

The Spearman correlation analysis conducted in the PHPT group revealed a significant positive correlation between depressive temperament and anxiety score (r = 0.638, p < 0.001), as well as between depressive temperament and depression score (r = 0.588, p < 0.001) (see Table 4). Cyclothymic temperament exhibited a significant correlation with anxiety (r = 0.587, p < 0.001) and with depression (r = 0.352, p = 0.015). Irritable temperament exhibited a significant positive correlation with anxiety (r = 0.580, p < 0.001) and with depression (r = 0.327, p = 0.025). Anxious temperament exhibited a significant correlation with anxiety (r = 0.769, p < 0.001) and with depression (r = 0.515, p < 0.001). Hyperthymic temperament exhibited no significant correlation with anxiety (r = 0.005, p = 0.972) or depression (r = −0.245, p = 0.097) scores.

**Table 4:** Correlations between temperament dimensions and anxiety/depression scores.

Group	TEMPS-A Subscale	HADS-Anxiety		HADS-Depression	
r	p	r	p
PHPT (n = 47)	Depressive	0.638	<0.001**	0.588	<0.001**
	Cyclothymic	0.587	<0.001**	0.352	0.015*
	Hyperthymic	0.005	0.972	−0.245	0.097
	Irritable	0.580	<0.001**	0.327	0.025*
	Anxious	0.769	<0.001**	0.515	<0.001**
Control (n = 36)	Depressive	−0.124	0.470	0.367	0.028*
	Cyclothymic	−0.082	0.634	0.155	0.367
	Hyperthymic	−0.182	0.289	−0.414	0.012*
	Irritable	−0.112	0.515	0.164	0.338
	Anxious	−0.094	0.586	0.326	0.048*

r: Spearman’s rank correlation coefficient; *p < 0.05; **p < 0.01*.

Correlation analyses in the control group revealed a significant positive correlation between depressive temperament and depression (r = 0.367, p = 0.028), as well as a correlation between anxious temperament and depression (r = 0.326, p = 0.048). A negative correlation was identified between hyperthymic temperament and depression scores (r = −0.414, p = 0.012). No significant relationship was identified between other temperament sub-dimensions and levels of depression or anxiety (all p > 0.05).

### The correlation between biological parameters and temperament

The correlation analyses performed in the PHPT group revealed a positive and significant association between hyperthymic temperament and serum calcium level (r = 0.371, p = 0.013). No significant correlation exists between other temperament dimensions and serum calcium levels (p > 0.05). A statistically significant relationship was not observed between PTH level and any temperament sub-dimension (all p > 0.05). No significant correlation was observed between serum TSH levels and temperament scores (all p > 0.05).

### Results of the generalized linear model (GLM)

To identify predictors of anxiety in patients with primary hyperparathyroidism, a generalized linear model (GLM) was developed using a Gaussian distribution and identity link function. The null model (H₀) included only the intercept, while the full model (H₁) incorporated all temperament sub-dimensions and biochemical variables. The deviance for the H₀ model was 703.909 (AIC = 250.855, BIC = 254.423), whereas the H₁ model showed a deviance of 257.365 (AIC = 212.584, BIC = 221.505). The reduction in AIC and BIC values indicated a superior fit for the H₁ model.

Backward elimination was subsequently performed, and the final model included three variables: cyclothymic temperament, anxious temperament, and serum calcium level. Among these, anxious temperament (β = 0.363, SE = 0.072, t = 5.010, p < 0.001, 95% CI: 0.221 to 0.505) and cyclothymic temperament (β = 0.210, SE = 0.094, t = 2.233, p = 0.031, 95% CI: 0.026 to 0.395) were statistically significant predictors of anxiety, while serum calcium showed a negative but not statistically significant association (β = −0.759, SE = 0.401, t = −1.893, p = 0.066, 95% CI: −1.545 to 0.027) (see Table 5).

**Table 5:** Predictors of anxiety score in phpt patients: generalized linear model results.

Variable	Estimate (β)	SE	t	p	95% CI (Lower–Upper)
Intercept	11.430	4.550	2.512	0.016*	2.512–20.348
Cyclothymic temper.	0.210	0.094	2.233	0.031*	0.026–0.395
Anxious temper.	0.363	0.072	5.010	<0.001**	0.221–0.505
Calcium (mg/dL)	−0.759	0.401	−1.893	0.066	−1.545–0.027

SE: Standard error; CI: Confidence interval; *p < 0.05; **p < 0.01*.

## Discussion

The present study examined the correlation between affective temperament traits and symptoms of depression and anxiety in individuals diagnosed with primary hyperparathyroidism (PHPT). Our findings indicated a significant association between anxious and cyclothymic temperament scores and anxiety levels in patients with primary hyperparathyroidism (PHPT). Although hyperthymic temperament scores and depression symptoms were found to be negatively correlated, this relationship was not statistically significant. These results indicate that the neuropsychiatric effects of primary hyperparathyroidism (PHPT) can differ among patients, with an individual’s inherent mood temperament potentially influencing the severity of psychological manifestations associated with the condition. These findings will be reviewed in light of the extant literature; similar or contradictory results will be evaluated, the study’s original contributions will be highlighted, and the implications for clinical and research practice will be discussed. This study is pioneering in its examination of psychiatric symptom levels in PHPT and the predictive effects of inherited temperament traits on these symptoms, utilizing multivariate statistical modeling. In this regard, it provides a novel contribution to the literature.

The examination of affective temperaments as predictors of psychiatric outcomes is expanding beyond psychiatric patient groups to include populations with medical diseases. The modulation of psychological responses to diseases by temperamental characteristics is a significant inquiry, particularly concerning chronic physical illnesses. A pilot study indicates that patients with fibromyalgia exhibit significantly elevated scores in depressive, cyclothymic, and anxious temperament compared to healthy controls [36]. Furthermore, symptoms of depression and anxiety are notably more prevalent in this patient group than in the general population. Furthermore, research indicates a positive correlation between HAD anxiety scores in fibromyalgia patients and cyclothymic as well as anxious temperament scores. Additionally, the severity of depression is linked to depressive and other temperament dimensions. Research on fibromyalgia indicates that affective temperament may serve as a predictor for the risk of depression and anxiety linked to chronic pain. The findings indicate that affective temperament profiles may influence the psychological outcomes associated with medical illnesses.

While a direct temperament study on PHPT is lacking, our findings align with existing literature on fibromyalgia. The primary finding indicates a significant correlation between anxious and cyclothymic temperaments in PHPT patients and their HAD anxiety scores. Individuals exhibiting anxious and fluctuating temperaments in fibromyalgia and primary hyperparathyroidism demonstrate heightened anxiety symptoms associated with their respective medical conditions [36]. This parallelism indicates that affective temperament profiles may similarly elevate the psychological burden experienced by patients across various chronic diseases [37,38]. Anxiety levels may rise disproportionately in metabolic stress situations, such as primary hyperparathyroidism (PHPT), particularly in individuals with an anxious temperament. Additionally, cyclothymic temperament can contribute to notable mood fluctuations, further exacerbating psychological variability. Previous studies have demonstrated that cyclothymic temperament is associated with increased vulnerability to anxiety and depressive symptoms, likely due to emotional instability and impulsivity [39,40]. The association between cyclothymic temperament and anxiety scores in our study indicates that stressors related to PHPT may elicit heightened anxiety responses in individuals exhibiting this temperament.

Our findings indicate a significant association between irritable temperament and levels of both anxiety and depression. This situation aligns with prior research reporting that irritable temperament is closely linked to affective instability, aggression, and increased mood disorder severity, including elevated anxiety and depressive symptoms [41,42]. In our analyses, irritable temperament showed significant correlations with both anxiety and depression in univariate models; however, this association was no longer significant in the multivariate GLM. This attenuation is likely due to the substantial shared variance between irritable temperament and other affective temperament dimensions, particularly anxious and cyclothymic traits, which demonstrated stronger and more specific independent effects in the multivariate model. When these overlapping predictors were included simultaneously, the unique contribution of irritable temperament diminished, a pattern consistent with previous temperament research where multicollinearity between affective traits commonly reduces independent predictive value. Therefore, the loss of significance in the adjusted model does not necessarily indicate a lack of clinical relevance, but rather reflects the stronger explanatory power of anxious and cyclothymic temperaments in this patient group.

An important methodological consideration in our study is the age difference between the PHPT and control groups. To account for this, we conducted age-adjusted GLM analyses. The results demonstrated that key findings including higher depressive, cyclothymic, irritable, and anxious temperament scores, as well as markedly elevated anxiety levels in PHPT patients, remained significant after controlling for age. Only the difference in HADS depression scores lost significance after adjustment. This suggests that while depressive symptoms may be partially age-related, the temperament profile and anxiety burden observed in PHPT are not attributable to age and therefore represent independent psychological features of the disorder.

This notable finding highlights the influence of emotional reactivity on the severity of mental symptoms in PHPT, suggesting that individuals with high cyclothymic or irritable temperament may be particularly susceptible to psychological distress in the context of endocrine stress.

The present study indicates that hyperthymic temperament may serve a protective function against symptoms of depression. Our findings indicate that patients with primary hyperparathyroidism exhibiting hyperthymic temperament scored lower on the hospital depression scale. The association between hyperthymic temperament and positive biological markers may be linked to biochemical factors influencing neurotransmission, including calcium. A significant positive correlation was identified between serum calcium levels and hyperthymic temperament in this study. This finding may be linked to the role of calcium in synaptic transmission and neurotransmitter release [43]. Previous reviews indicate that hyperthymic temperament is associated with a decreased risk of suicide and offers resilience against depressive and anxious symptoms [41]. Consequently, it is reasonable to conclude that their psychological state remains relatively intact when confronted with biological stress induced by PHPT. The literature indicates that hyperthymic temperament correlates with a decreased risk of suicide and increased resilience against depressive and anxious symptoms [39,44,45]. Hyperthymic individuals, characterized by elevated energy, vitality, optimism, and stress-coping abilities, exhibit greater psychological resilience and protection against biologically stressful conditions like PHPT.

These findings reflect two distinct analytical levels and therefore do not represent a contradiction. At the between-group level, hyperthymic temperament does not appear to be elevated or reduced in individuals with PHPT compared with healthy controls, indicating that the condition itself does not selectively influence overall hyperthymic trait levels. In contrast, at the within-group level, variation in hyperthymic temperament among PHPT patients shows a meaningful pattern: individuals with relatively higher hyperthymic scores tend to report lower depressive symptoms. This suggests that while hyperthymic traits are not characteristic of PHPT as a disorder, they may still play a protective role in modulating depressive symptom severity among patients who already have PHPT.

Conversely, after multivariate adjustment, irritable temperament did not exhibit a statistically significant independent relationship, whereas depressive temperament, despite showing significant univariate correlations, was not retained as an independent predictor in the final model. High depressive temperament is typically linked to the onset of major depression, suggesting that it may influence current depression scores in patients with PHPT [41]. Nevertheless, the absence of a significant relationship between depressive temperament and HAD depression scores in our cases may indicate that depression associated with PHPT is influenced more by situational and biochemical factors, whereas temperamental sensitivity is more relevant to the anxiety dimension. The restricted sample size may have hindered the statistical significance of certain relationships. A comparable scenario may apply to irritable temperament; while this temperament type is characterized in the literature as a trait that elevates suicide risk and is linked to the bipolar spectrum [46,47], irritability did not show a significant correlation with symptoms of depression or anxiety in our PHPT series. This indicates that irritable temperament may not be significant in the context of PHPT or may have intersected with other measured temperament factors, such as cyclothymia.

Taken together, the present study makes several novel contributions to the literature. First, it is among the pioneering efforts to systematically examine the relationship between affective temperament profiles and neuropsychiatric manifestations in primary hyperparathyroidism, thereby bridging the fields of endocrinology and psychiatry through an integrative, biopsychosocial perspective. Second, our multivariate modeling approach demonstrates that specific temperament traits—particularly anxious and cyclothymic—serve as significant predictors of anxiety symptoms, while hyperthymic temperament may act as a protective factor against depression. Third, by incorporating temperament assessment (e.g., with the TEMPS-A scale) into the evaluation of PHPT patients, clinicians may be better equipped to identify individuals at heightened risk for psychiatric comorbidities and tailor interventions proactively. These findings suggest that routine temperament profiling could enhance clinical risk stratification in PHPT, representing a practical and innovative addition to current patient management strategies. Finally, our study is one of the first to directly assess the predictive value of multiple affective temperament dimensions within a single multivariate model in a medical disease context, supporting the utility of a comprehensive approach that integrates both biological and personality-based factors in psychiatric assessment.

These findings have several important implications for clinical practice. First, psychiatric symptom screening should be routinely integrated into the assessment of patients with PHPT, even in cases of mild hypercalcemia, as depression and anxiety may be overlooked in these patients [48]. Identification of anxious and cyclothymic temperament profiles can help clinicians anticipate a higher psychiatric burden and guide timely intervention and follow-up. Although hyperthymic temperament may confer relative resilience, continued psychosocial assessment remains necessary for all patients. Furthermore, consideration of severe psychiatric involvement in PHPT could strengthen the indication for parathyroidectomy, as suggested in recent surgical guidelines [49,50].

In terms of research, this study opens new avenues for understanding the neuropsychiatric dimensions of PHPT. Prospective, longitudinal studies are needed to determine how affective temperament traits influence the long-term psychiatric trajectory and treatment response in PHPT. For example, it remains to be seen whether anxious temperament predicts greater benefit or persistent vulnerability following parathyroidectomy, and whether hyperthymic temperament is associated with more rapid improvement in depressive symptoms. Future research should also examine whether affective temperament has a similar predictive role in other endocrine disorders and investigate the neurobiological mechanisms underlying these interactions using neuroimaging and neurochemical studies.

### Study limitations

As well as its advantages, this study contains limitations. The sample size was limited and derived from a single-center patient group; thus, caution is warranted concerning the generalizability of the findings. Additionally, given the cross-sectional nature of the study design, it is crucial to note that the observed correlations do not allow for definitive conclusions regarding causality. Higher anxiety scores in patients with anxious temperaments may suggest that PHPT exacerbates anxiety in these individuals; conversely, the anxiety induced by PHPT may also amplify the patients’ anxious temperament. In the absence of longitudinal studies, establishing the direction of this interaction is challenging. We evaluated levels of depression and anxiety utilizing the Hospital Anxiety Depression Scale (HADS). This self-report scale is beneficial for screening chronic illnesses as it omits somatic symptoms; however, it does not yield a psychiatric diagnosis. Future studies utilizing diagnoses confirmed through clinical interviews, along with control groups when necessary, will elucidate the relationship between temperament and psychopathology more clearly.

In addition to the limitations noted above, several methodological considerations should be acknowledged. First, both temperament (TEMPS-A) and psychiatric symptom levels (HADS) were assessed using self-report instruments, which may be subject to response bias, including social desirability and recall bias. Although these measures are widely used and validated, future research incorporating clinician-administered assessments may help reduce potential measurement bias. Second, our sample consisted predominantly of female participants, which may limit the generalizability of the findings to males with PHPT. Given documented gender differences in affective temperament profiles and psychiatric symptom expression, studies with more balanced gender distributions are needed to confirm these results. Third, the control group was recruited from individuals attending an endocrinology clinic for general health screening, rather than from a community-based sample. Although they were medically healthy, this recruitment strategy may introduce selection bias and limit the representativeness of the control group. Future studies involving population-based controls would help strengthen the external validity of the findings.

## Conclusions

Affective temperament characteristics may significantly influence the severity of depression and anxiety symptoms in patients with primary hyperparathyroidism. Individuals exhibiting anxious and cyclothymic temperaments endure a more pronounced psychological burden associated with PHPT, whereas those with hyperthymic temperaments seem to possess a degree of protection against this burden. These results highlight the necessity of integrating the psychological characteristics of each patient with their biological factors in the management of primary hyperparathyroidism (PHPT). This study provides a novel perspective on the existing debate in the literature concerning the neuropsychiatric effects of primary hyperparathyroidism (PHPT), emphasizing the significance of endopsychiatric approaches. Future research must seek to establish these relationships causally through larger samples and extended follow-up data, while also enhancing patient care by integrating these findings into clinical guidelines. This approach will render the often overlooked psychological burden of primary hyperparathyroidism (PHPT) apparent, facilitating the formulation of treatment strategies that encompass both physical and mental health for affected individuals. Temperament screening through brief self-report scales like the TEMPS-A could serve as a practical method in clinical settings for individualized risk assessment and early intervention. This could ease the identification of high-risk groups among PHPT patients and the formulation of specific psychiatric support and follow-up strategies.

## Supporting information

S1 Table Demographic and clinical characteristics of the study sample by affective temperament groups. (DOCX)
