# Supplementary material for: The role of affective temperaments in predicting depression and anxiety symptoms in patients with primary hyperparathyroidism
Source: PLoS One. 2025 Dec 26;20(12):e0339321. doi: 10.1371/journal.pone.0339321 (PMC12742794; doi:10.1371/journal.pone.0339321)
Supplement: S1 Table — (DOCX) [file pone.0339321.s001.docx]

**S1 Table. Age-Adjusted General Linear Models (GLM)**

| Outcome | Group coef. | Group p | Age coef. | Age p |
| --- | --- | --- | --- | --- |
| HADS Anxiety | -7.8367 | 1.37E-14 | -0.0476 | 0.111 |
| HADS Depression | -2.0914 | 0.052 | 0.0135 | 0.720 |
| Depressive temperament | -5.0938 | 1.57E-06 | -0.031 | 0.377 |
| Cyclothymic temperament | -3.793 | 0.0027 | -0.0673 | 0.014 |
| Hyperthymic temperament | 0.8819 | 0.465 | 0.0016 | 0.970 |
| Irritable temperament | -3.6727 | 0.00059 | -0.0963 | 0.0098 |
| Anxious temperament | -6.2698 | 0.00028 | -0.0783 | 0.184 |

**GLM: Generalized Linear Model; HADS: Hospital Anxiety and Depression Scale; TEMPS-A: Temperament Evaluation of Memphis, Pisa, Paris and San Diego Auto-questionnaire.
Table shows group (PHPT vs. control) coefficients and age-adjusted effects for each outcome. Negative group coefficients indicate higher scores in the PHPT group after controlling for age.**
